# The feasibility of a heart block with an electron compensation as an alternative whole breast radiotherapy technique in patients with underlying cardiac or pulmonary disease

**DOI:** 10.1371/journal.pone.0184137

**Published:** 2017-09-01

**Authors:** Hye Jin Kang, Shin-Wook Kim, Seok Hyun Son

**Affiliations:** Department of Radiation Oncology, Incheon St. Mary's Hospital, College of Medicine, The Catholic University of Korea, Seoul, Republic of Korea; Northwestern University Feinberg School of Medicine, UNITED STATES

## Abstract

**Purpose:**

We aimed to evaluate the feasibility of the heart block with electron compensation (HBE) technique, based on three-dimensional conformal radiotherapy (3D-CRT) in left-sided breast cancer patients with underlying cardiac or pulmonary disease.

**Methods:**

Twenty patients with left-sided breast cancer who were treated with whole breast radiotherapy (WBRT) were included in this study. Intensity-modulated radiotherapy (IMRT), 3D-CRT, and HBE treatment plans were generated for each patient. Based on the 3D-CRT plan, the HBE plan included a heart block from the medial tangential field to shield the heart and added an electron beam to compensate for the loss in target volume coverage. The dosimetric parameters for the heart and lung and the target volume between the three treatment types were compared.

**Results:**

Of the three plans, the HBE plan yielded the most significant reduction in the doses received by the heart and lung (heart Dmean: 5.1 Gy vs. 12.9 Gy vs. 4.0 Gy and lung Dmean: 11.4 Gy vs. 13.2 Gy vs. 10.5 Gy, for 3D-CRT, IMRT, and HBE, respectively). Target coverage with all three techniques was within the acceptable range (Dmean 51.0 Gy vs. 51.2 Gy vs. 50.6 Gy, for 3D-CRT, IMRT, and HBE, respectively).

**Conclusions:**

The HBE plan effectively reduced the amount of radiation exposure to the heart and lung. It could be beneficial for patients who are vulnerable to radiation-related cardiac or pulmonary toxicities.

## Introduction

Breast conserving surgery followed by whole breast radiotherapy is the standard treatment for patients with early stage breast cancer; it improves local control and overall survival [1–3]. However, exposure of organs at risk (OARs) in the thorax, such as the heart and lungs, to high dose radiation is inevitable in patients treated with three-dimensional conformal radiotherapy (3D-CRT), the most commonly used technique. The radiation dose delivered to OARs increases as the size of the breast and the distance to the chest wall increases [4, 5]. In particular, patients with left-sided breast cancer have an increased risk of radiation-related cardiac toxicity [6]. The mortality risk from normal tissue exposure to radiation offsets the survival benefits gained by radiotherapy [2]. Therefore, radiation-related toxicities, as well as cancer control, should be considered when determining the treatment strategies for breast cancer.

Numerous studies have observed the dosimetric benefit of multiple-field intensity-modulated radiotherapy (IMRT) compared with conventional 3D-CRT for whole breast radiotherapy [7–10]. These studies reported improved target coverage and reduced high-dose volumes to the heart and lungs; however, these approaches forfeited the larger volume exposure achieved with the low-to-moderate doses used in the IMRT treatment plans. The clinical importance of low-to-moderate dose exposure to the heart and lungs in terms of radiation-related toxicities has been well studied. The incidence of life-threatening toxicities is relatively low in patients with intact cardiac and pulmonary functions. However, these toxicities could be fatal for patients with underlying cardiac or pulmonary diseases such as ischemic heart disease, interstitial lung disease, or a history of anatomic lung resection [11–15].

In this study, we propose an alternative whole breast radiotherapy technique for these vulnerable patients. Based on the 3D-CRT plan, we added an ipsilateral heart block to the medial tangential field to reduce the dose delivered to the heart, and added an electron beam to compensate the target volume coverage, which is compromised by a heart block. To evaluate the feasibility of this technique, we performed a dosimetric comparison of the three treatment plans: 3D-CRT vs. IMRT vs. heart block with electron compensation based on 3D-CRT (HBE). We also evaluated the target volume coverage as well as the heart and lung dose-sparing effect in left-sided breast cancer patients with underlying cardiac or pulmonary disease.

## Materials and methods

### Patient characteristics, CT simulation, delineation of target volume, and OARs

Twenty patients who were diagnosed with early stage left-sided breast cancer with negative axillary lymph nodes between November 2014 and May 2015 were selected for this study. Patient characteristics were presented in Table 1. All of the patients underwent whole breast radiotherapy using the 3D-CRT technique after breast conserving surgery. Their computed tomography (CT) scans were evaluated. Additional IMRT and HBE plans were performed for this comparative study following institutional review board approval (IRB of Incheon St. Mary’s Hospital, College of Medicine, The Catholic University of Korea, Reference number: OC16RASI0112). IRB approved that this study was exempted from obtaining of written informed consent because of the retrospective nature of this study.

**Table 1:** Clinical characteristics of 20 patients.

Patient no.	Age (year)	Height (cm)	Weight (kg)	BSA (m^2^)	pT stage	Tumor location	PTV (cm^3^)
1	50	162.9	56.0	1.60	T2	UIQ	270.4
2	50	152.3	59.9	1.56	Tis	LOQ	509.1
3	42	157.6	67.2	1.68	T2	UIQ	231.1
4	59	159.0	48.3	1.47	T1	UOQ	111.1
5	62	157.5	53.1	1.52	T1	UOQ	475.3
6	50	155.0	55.5	1.53	T1	UOQ	547.9
7	51	161.8	62.5	1.66	Tis	LOQ	451.2
8	55	146.8	52.5	1.44	T2	UIQ	401.2
9	46	158.0	59.4	1.60	T1	LIQ	262.4
10	47	155.5	70.0	1.70	T2	UOQ	585.6
11	60	152.0	56.8	1.53	Tis	UOQ	508.7
12	44	164.6	59.1	1.65	T1	UOQ	474.2
13	46	160.0	43.2	1.41	T1	LIQ	144.3
14	52	154.6	55.7	1.53	T1	UOQ	444.1
15	52	151.2	52.9	1.48	T1	UIQ	659.3
16	53	157.1	54.5	1.54	T1	UOQ	419.8
17	44	151.5	57.6	1.53	Tis	UOQ	256.9
18	44	156.0	56.0	1.55	T1	UOQ	395.7
19	47	159.3	59.3	1.61	Tis	UIQ	333.1
20	62	142.0	51.0	1.39	T1	UOQ	471.0

Abbreviation: BSA = body surface area; UIQ = upper inner quadrant; UOQ = upper outer quadrant; LIQ = lower inner quadrant; LOQ = lower outer quadrant; PTV = planning target volume.

The selected patients were placed in the supine position on a breast-tilting board with a 10° angle with both arms placed above their heads. Prior to CT scanning, palpitation of the breast was performed to identify the extent of the breast parenchyma and the breast skin was marked using radio-opaque non-metallic wire for target volume delineation. CT images were acquired using a LightSpeed RT16 CT scanner (GE Healthcare, Waukesha, WI). The slice thickness was 2.5 mm, with complete coverage of the heart, both lungs, and bilateral breasts. The CT images were sent to Eclipse version 8.9 (Varian Medical Systems, Palo Alto, CA) via a Digital Imaging and Communications in Medicine network connection.

For consistency, delineation of all contours was performed by one radiation oncologist. The planning target volume (PTV) was delineated in accordance with European Society for Radiotherapy & Oncology guidelines [16]. PTV included the whole breast parenchyma that was distinguishable on the simulation CT and wired through palpation. The heart was delineated from the inferior side of the pulmonary artery to the cardiac apex along the pericardial space. The contours of both lungs were automatically created by the Eclipse software.

### Design of treatment plans

For consistency, a single radiation physicist carried out all of the planning procedures. In the 3D-CRT plan, two opposing tangential fields were created as the main fields. The gantry angles and beam weights were individually optimized for adequate treatment. This plan used a 6 MV photon beam, and the analytic anisotropic algorithm (version 8.9.17) with a grid size of 2.5 mm was used for the dose calculation. To enhance the dose homogeneity, lateral wedge was used only when necessary. The superior and inferior borders of the field were set at the suprasternal notch and 2 cm below the inframammary fold, respectively. The medial border of the field was the patient’s midline. Each plan was normalized to a reference point located between the breast and the pectoralis major muscle at the nipple level. We used the field-in-field technique to attenuate hot spots and improve the homogeneity of the PTV. This plan had 1–4 subfields.

**Fig 1:**
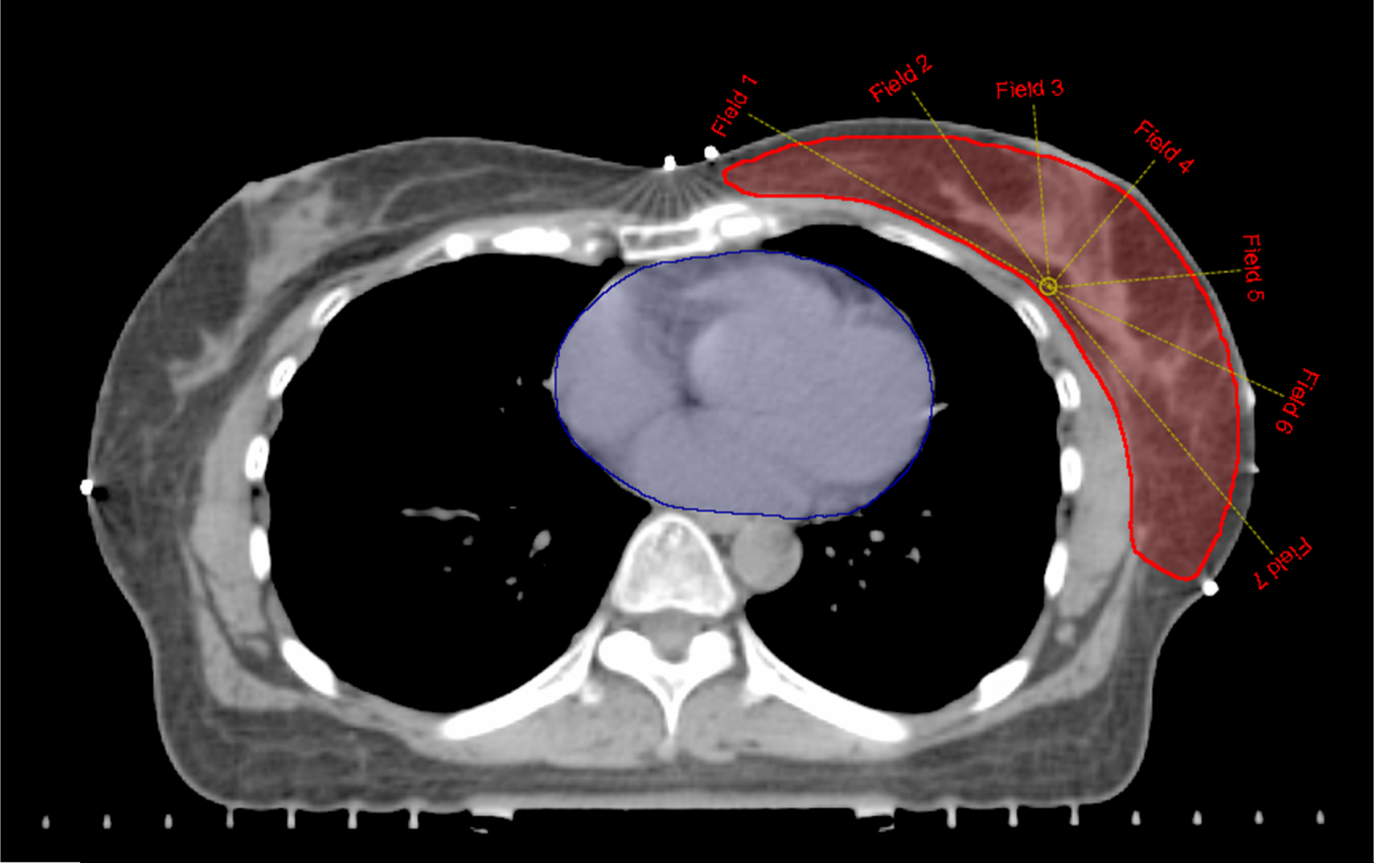
Beam arrangement for the IMRT technique.

An IMRT plan was developed using an inverse treatment planning technique. The IMRT plan consisted of seven fields in a hinge angle of 200° that was generated by adjusting the gantry angle by -10° from the medial tangential beam and +10° from the lateral tangential beam. The beam arrangement intervals were (clockwise) 45°, 30°, 20°, 20°, 30°, and 45° from the medial to the lateral beam (Fig 1). The photon energy and the algorithm for the dose calculation were the same as those used in the 3D-CRT plan. The IMRT plans were developed to deliver 95% of the prescribed dose to the entire PTV. We tried to minimize the irradiation dose to the heart and lungs, while still maintaining acceptable PTV coverage. All IMRT plans were generated with the same dose constraints and number of iterations.

The HBE plan was developed by modifying the 3D-CRT plan. In order to prevent high-dose radiation exposure to the heart regions within the tangential fields, we added a block from the medial tangential field to shield the heart using a multileaf collimator on the beam’s eye view. This heart block resultantly compromised the PTV coverage. To compensate for this compromised PTV coverage, an electron beam was added to the shielded area. The shape of the electron beam shape was such that it completely overlapped with the heart block on the beam’s eye view of the target. The course of the electron beam was selected so that it was medially tilted 5° from the medial tangential beam. The virtual source to skin distance for the electron beam was 100 cm. The electron energy of 6, 9, or 12 MeV was chosen depending on the depth of the PTV. Electron Monte Carlo (version 8.9.08) with a grid size of 5 mm was used to calculate the electron dose.

**Fig 2:**
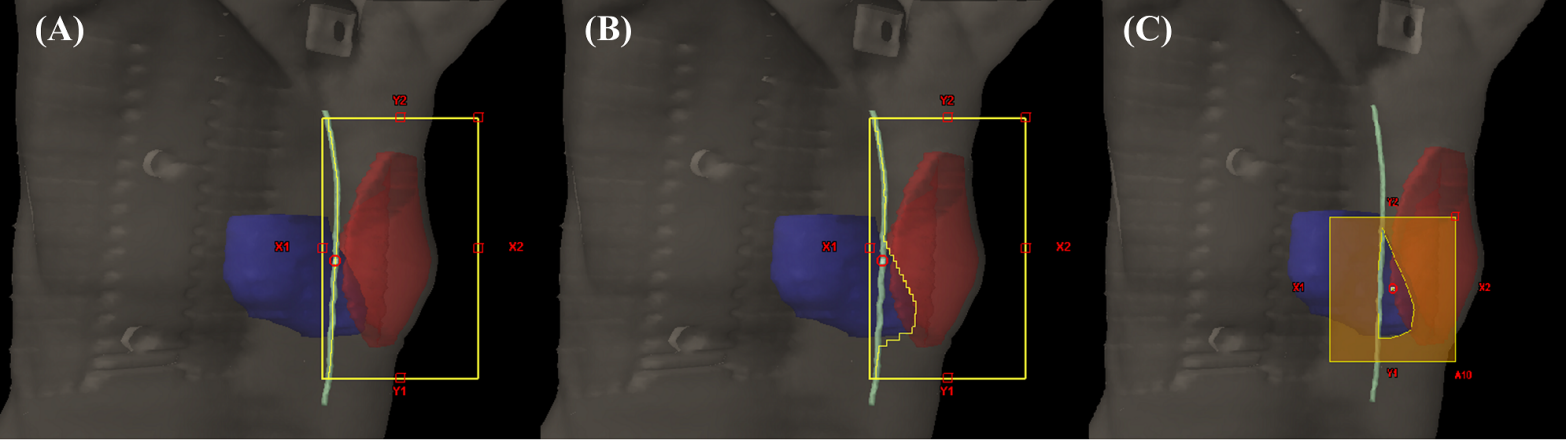
Beam’s eye views of the HBE plans. (A) The medial tangential field of the 3D-CRT plan was the base of the HBE plan. The medial border of the field was the patient’s midline; (B) The medial tangential field of the HBE plan was designed by blocking the shape of the heart; (C) The electron beam of the HBE plan was completely overlapped with the heart block on the beam’s eye view at an angle of 5° (PTV = red area; heart = blue area; midline = light green line). (Abbreviation: HBE = heart block with electron compensation radiotherapy; PTV = planning target volume; 3D-CRT = three-dimensional conformal radiotherapy).

To align the junction of the photon and electron beams in this planning process, the electron field was based on the patient’s midline and the medial border of the tangential photon beam. This process is presented in Fig 2. In actual patient treatment, the electron field is similarly set up between the midline and the medial border of the tangential photon field, which is obtained by turning on the beam light after positioning.

### Comparison of plans and statistical analysis

In order to valid comparison of the dose distribution between plans, dose-volume histograms (DVHs) of the 3D-CRT plan and the HBE plans were set as D50 (the minimum dose for 50% of target volume) of the PTV on IMRT plan in each patient. Three types of treatment plan were compared by assessment of dosimetric parameters. To compare the cardiac sparing effect, V10 (percentage of volume receiving more than 10 Gy), V20, V30, V40, V45, and Dmean (mean dose) were estimated. For the ipsilateral lung, V5, V10, V15, V20, and Dmean were assessed. And for the PTV, V40, V45, V47.5, V50, and Dmean were reported.

Dosimetric parameters between plans were compared using the Kruskal–Wallis test, and multiple comparison tests were performed with the Wilcox signed-rank tests with *p-*values adjusted using the Benjamini & Hochberg correction method. In order to determine the possible association between the dose to OARs and physical condition of patient [17–19], a correlation analysis was used for each treatment plan. All the test results were two-sided. A *p*-value of < 0.05 was considered statistically significant. All statistical analyses were done with R version 3.2.1 (R Development Core Team, Vienna, Austria).

## Results

### Dose distribution of the OARs and PTV

**Fig 3:**
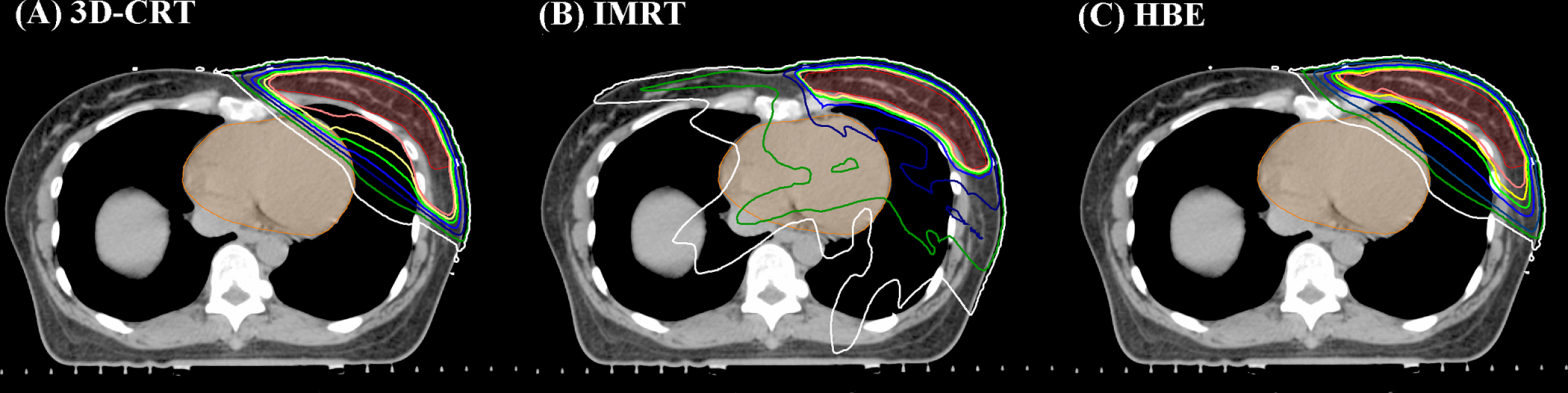
**Dose distributions of (A) the 3D-CRT, (B) IMRT and (C) HBE plans** (PTV = semi-lucent red area; heart = semi-lucent orange area; pink line = 49 Gy; yellow line = 47.5 Gy; yellowish green line = 45 Gy; blue line = 40 Gy; navy line = 25 Gy; green line = 10 Gy; white line = 5 Gy). (Abbreviation: 3D-CRT = three-dimensional conformal radiotherapy; HBE = heart block with electron compensation radiotherapy; IMRT = intensity-modulated radiotherapy; PTV = planning target volume).

**Fig 4:**
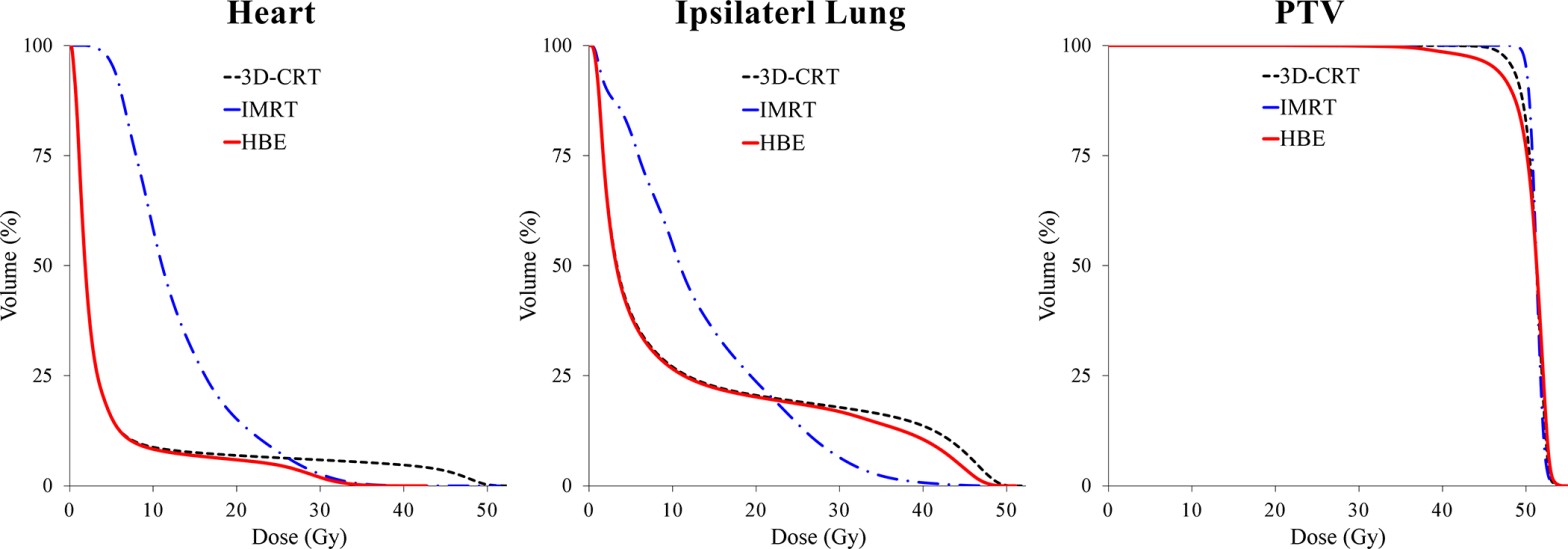
Comparison of dose–volume histograms for heart, ipsilateral lung and PTV between plans. (Abbreviation: PTV = planning target volume; 3D-CRT = three-dimensional conformal radiotherapy; HBE = heart block with electron compensation radiotherapy; IMRT = intensity-modulated radiotherapy).

The dosimetric parameters for the OARs and PTV in the three treatment plans are summarized in Table 2. All values are presented as mean dose ± standard deviation. Dose distributions between the three plans for a representative patient are displayed (Fig 3), and the average cumulative DVHs of the heart, ipsilateral lung, and PTV for all patients are shown in (Fig 4).

**Table 2:** Dosimetric comparison for OARs between 3D-CRT, HBE and IMRT.

Structure	Parameters	3D-CRT	IMRT	HBE	*p*-value
Heart	V10 (%)	8.8 ± 3.5	58.2 ± 10.6	8.3 ± 3.3	*<0*.*001*
	V20 (%)	6.9 ± 3.1	15.2 ± 6.1	5.9 ± 2.7	*<0*.*001*
	V30 (%)	5.9 ± 2.8	2.7 ± 2.1	1.9 ± 1.1	*<0*.*001*
	V40 (%)	4.7 ± 2.4	0.1 ± 0.1	0.0 ± 0.0	*<0*.*001*
	V45 (%)	3.4 ± 1.9	0.0 ± 0.0	0.0 ± 0.0	*<0*.*001*
	Dmean (Gy)	5.1 ± 1.6	12.9 ± 1.6	4.0 ± 1.0	*<0*.*001*
Ipsilateral lung	V5 (%)	39.3 ± 5.0	80.2 ± 6.4	38.6 ± 5.1	*<0*.*001*
	V10 (%)	27.0 ± 4.5	54.9 ± 4.5	26.6 ± 4.5	*<0*.*001*
	V15 (%)	22.6 ± 4.3	35.2 ± 4.4	22.3 ± 4.3	*<0*.*001*
	V20 (%)	20.6 ± 4.2	23.7 ± 4.6	20.2 ± 4.2	*0*.*026*
	V40 (%)	13.6 ± 3.7	0.7 ± 0.8	10.5 ± 3.8	*<0*.*001*
	Dmean (Gy)	11.4 ± 1.7	13.2 ± 1.1	10.5 ± 2.6	*<0*.*001*
Total lung	V5 (%)	17.3 ± 2.8	44.0 ± 4.4	17.1 ± 2.8	*<0*.*001*
	V10 (%)	12.0 ± 2.5	24.2 ± 2.1	11.8 ± 2.4	*<0*.*001*
	V20 (%)	9.1 ± 2.2	10.4 ± 2.2	9.0 ± 2.2	*0*.*074*
	Dmean (Gy)	5.3 ± 2.2	7.7 ± 0.6	5.0 ± 0.9	*<0*.*001*
PTV	V40 (%)	99.9 ± 0.1	100.0 ± 0.0	98.5 ± 2.4	*<0*.*001*
	V45 (%)	99.7 ± 0.3	100.0 ± 0.0	96.3 ± 4.7	*<0*.*001*
	V47.5 (%)	97.3 ± 1.0	100.0 ± 0.0	92.2 ± 5.9	*<0*.*001*
	V50 (%)	82.6 ± 4.1	95.0 ± 0.0	76.5 ± 6.3	*<0*.*001*
	Dmean (Gy)	51.0 ± 0.2	51.2 ± 0.2	50.6 ± 0.6	*<0*.*001*

PTV: planning target volume; 3D-CRT: three-dimensional conformal radiotherapy; HBE: heart block with electron compensation radiotherapy; IMRT: intensity-modulated radiotherapy; V*x*: volume (%) receiving at least *x* (Gy) dose; Dmean: mean dose.

In terms of the dose delivered to the heart, the IMRT plan effectively decreased the higher doses by more than 30 Gy compared with the 3D-CRT plan (V30: 5.9 ± 2.8 Gy vs. 2.7 ± 2.1 Gy; V40: 4.7 ± 2.4 Gy vs. 0.1 ± 0.1 Gy, for the 3D-CRT and IMRT plans, respectively). However, the target volume reached with the low-to-moderate dose and the mean dose were higher with the IMRT plan than with the 3D-CRT plan (V10: 8.8 ± 3.5 Gy vs. 58.2 ± 10.6 Gy; V20: 6.9 ± 3.1 Gy vs. 15.2 ± 6.1 Gy; Dmean: 5.1 ± 1.6 Gy vs. 12.9 ± 1.6 Gy, for the 3D-CRT and IMRT plans, respectively). The HBE plan resulted in significantly reduced high doses to the heart, similar to those achieved with the IMRT plan (V30: 5.9 ± 2.8 Gy vs. 2.7 ± 2.1 Gy vs. 1.9 ± 1.1 Gy; V40: 4.7 ± 2.4 Gy vs. 0.1 ± 0.1 Gy vs. 0.0 ± 0.0 Gy, for the 3D-CRT, IMRT, and HBE plans, respectively). In addition, the HBE plan did not increase the volume that received the low-to-moderate dose or the mean dose (V10: 8.8 ± 3.5 Gy vs. 58.2 ± 10.6 Gy vs. 8.3 ± 3.3 Gy; V20: 6.9 ± 3.1 Gy vs. 15.2 ± 6.1 Gy vs. 5.9 ± 2.7 Gy; Dmean: 5.1 ± 1.6 Gy vs. 12.9 ± 1.6 Gy vs. 4.0 ± 1.0 Gy, for the 3D-CRT, IMRT, and HBE plans, respectively).

With regard to the ipsilateral lung, the volume receiving the low-to-moderate dose and the mean dose was increased with the IMRT plan compared with the 3D-CRT or HBE plans (V5: 39.3 ± 5.0 Gy vs. 80.2 ± 6.4 Gy vs. 38.6 ± 5.1 Gy; V10: 27.0 ± 4.5 Gy vs. 54.9 ± 4.5 Gy vs. 26.6 ± 4.5 Gy; Dmean: 11.4 ± 1.7 Gy vs. 13.2 ± 1.1 Gy vs. 10.5 ± 2.6 Gy, for the 3D-CRT, IMRT, and HBE plans, respectively). In the HBE plan, the low-to-moderate dose area was similar to that in the 3D-CRT plan, and the high dose area was also slightly reduced because the heart block in HBE plan spared the ipsilateral lung (V40: 13.6 ± 3.7 Gy vs. 10.5 ± 3.8 Gy, for the 3D-CRT and HBE plans, respectively).

The mean PTV was 398 cm^3^ (range, 111–659 cm^3^). Not surprisingly, the IMRT plan demonstrated a significantly better dose coverage than the 3D-CRT or HBE plans (V45: 99.7 ± 0.3 Gy vs. 100.0 ± 0.0 Gy vs. 96.3 ± 4.7 Gy; V47.5: 97.3 ± 1.0 Gy vs. 100.0 ± 0.0 Gy vs. 92.2 ± 5.9 Gy; Dmean: 51.0 ± 0.2 Gy vs. 51.2 ± 0.2 Gy vs. 50.6 ± 0.6 Gy, for the 3D-CRT, IMRT, and HBE plans, respectively).

### Correlation between morphological factors and doses to the OARs

In terms of correlation between breast volume and dose to the OARs, there was no significant correlation between breast volume and radiation dose delivered to the heart in all three plans (3D-CRT vs. V10, r = 0.28, p = 0.24; 3D-CRT vs. V20, r = 0.28, p = 0.24; 3D-CRT vs. V30, r = 0.28, p = 0.24; 3D-CRT vs. V40, r = 0.28, p = 0.24; 3D-CRT vs. mean heart dose, r = 0.28, p = 0.22; IMRT vs. V10, r = 0.34, p = 0.14; IMRT vs. V20, r = 0.40, p = 0.18; IMRT vs. V30, r = 0.36, p = 0.12; IMRT vs. V40, r = -0.18, p = 0.46; IMRT vs. mean heart dose, r = 0.40, p = 0.07; HBE vs. V10, r = 0.28, p = 0.24; HBE vs. V20, r = 0.27, p = 0.26; HBE vs. V30, r = 0.11, p = 0.66; HBE vs. V40, r = -0.38, p = 0.10; HBE vs. mean heart dose, r = 0.26, p = 0.27). In case of lung dose, there was no significant correlation with the breast volume (3D-CRT vs. V10, r = 0.13, p = 0.59; 3D-CRT vs. V20, r = 0.13, p = 0.59; 3D-CRT vs. V30, r = 0.13, p = 0.55; 3D-CRT vs. V40, r = 0.17, p = 0.48; 3D-CRT vs. mean lung dose, r = 0.16, p = 0.50; IMRT vs. V10, r = 0.34, p = 0.15; IMRT vs. V20, r = 0.40, p = 0.08; IMRT vs. V30, r = 0.31, p = 0.18; IMRT vs. V40, r = 0.08, p = 0.74; IMRT vs. mean lung dose, r = 0.43, p = 0.06; HBE vs. V10, r = 0.12, p = 0.62; HBE vs. V20, r = 0.12, p = 0.60; HBE vs. V30, r = 0.12, p = 0.61; HBE vs. V40, r = 0.10, p = 0.67; HBE vs. mean lung dose, r = -0.02, p = 0.93)

In relation to body surface area, we found a statistically significant correlation between body surface area and dose to the heart in all three plans (3D-CRT vs. V10, r = 0.84, p < 0.01; 3D-CRT vs. V20, r = 0.83, p < 0.01; 3D-CRT vs. V30, r = 0.83, p < 0.01; 3D-CRT vs. V40, r = 0.82, p < 0.01; 3D-CRT vs. mean heart dose, r = 0.86, p < 0.01; IMRT vs. V10, r = 0.64, p < 0.01; IMRT vs. V20, r = 0.71, p < 0.01; IMRT vs. V30, r = 0.54, p < 0.01; IMRT vs. V40, r = 0.89, p = 0.03; IMRT vs. mean heart dose, r = 0.72, p < 0.01; HBE vs. V10, r = 0.76, p < 0.01; HBE vs. V20, r = 0.72, p < 0.01; HBE vs. V30, r = 0.46, p < 0.04; HBE vs. V30, r = -0.27, p = 0.24, HBE vs. mean heart does, r = 0.81, p < 0.01). However, no significant correlation exists between body surface area and dose to the lung (3D-CRT vs. V10, r = -0.15, p = 0.54; 3D-CRT vs. V20, r = -0.12, p = 0.63; 3D-CRT vs. V30, r = -0.11, p = 0.65; 3D-CRT vs. V40, r = -0.10, p = 0.67; 3D-CRT vs. mean lung does, r = -0.08, p = 0.71; IMRT vs. V10, r = 0.40, p = 0.08; IMRT vs. V20, r = 0.40, p = 0.08; IMRT vs. V30, r = 0.26, p = 0.27; IMRT vs. V40, r = 0.03, p = 0.89; IMRT vs. mean lung does, r = 0.40, p = 0.07; HBE vs. V10, r = -0.19, p = 0.41; HBE vs. V20, r = -0.18, p = 0.45; HBE vs. V30, r = -0.20, p = 0.39; HBE vs. V40, r = -0.27, p = 0.25; HBE vs. mean lung does, r = -0.28, p = 0.23)” (highlighted in red, page 10, lines 208–216).

## Discussion

A number of randomized controlled trials established that breast conserving surgery followed by radiotherapy and mastectomy had similar effects on survival [20, 21]. Although adjuvant radiotherapy following breast conserving surgery is generally preferred, mastectomy represents another treatment option for patients with severe underlying cardiac or pulmonary disease, because these patients are at high risk for developing radiation-related toxicities. Mastectomy in these vulnerable patients may not always dispense with the need for radiotherapy. In a population-based study, 22.4% of patients undergoing mastectomy had an indication for adjuvant radiotherapy [22]. If the irradiation dose to the critical organs can be effectively reduced compared with commonly used techniques such as 3D-CRT or IMRT, an unnecessary or undesirable mastectomy could be avoided. In this study, we devised an alternative whole breast radiotherapy technique, the HBE plan, and evaluated the feasibility of this technique.

Radiation-related cardiac toxicity is a complicated process involving different heart structures with different radiosensitivities [10, 23, 24]. Therefore, unavoidable controversy exists regarding the important dosimetric factors that can predict cardiac toxicity. Gagliardi *et al*. used a normal tissue complication probability model to predict excessive cardiac mortality in patients treated with whole breast radiotherapy and reported that the cardiac mortality risk increased rapidly with more than 30 Gy [25, 26]. Hardenbergh *et al*. reported the occurrence of heart dose-dependent myocardial perfusion defects from a single photon emission CT during a 6-month follow-up period after radiotherapy [27]. Cardiac areas that receive less than 10 Gy showed minimal defects, while regional perfusion was reduced by 20% when more than 40 Gy was applied. Recent studies also suggest a linear relationship between the mean heart dose and the incidence rates of major coronary events [14, 28]. The risk was increased by 7.4% per gray, with no apparent threshold. In addition, compared with patients with no underlying cardiac or pulmonary disease, the ratios of the rates of major coronary events in patients with ischemic heart disease history and chronic obstructive pulmonary disease were 1:6.67 and 1:6.33, respectively [14]. Therefore, it is recommended that the heart volume receiving more than 30 Gy and the mean dose should be minimized to prevent radiation-related cardiac toxicity, particularly in patients who are predisposed to cardiac disease. The major drawback of 3D-CRT, which is a widely used technique, is that areas of the heart are exposed to high doses of radiation. In this study, the heart volume that received more than 30 Gy was significantly lower with the IMRT plan compared with the 3D-CRT plan, while expansion of the low-to-moderate dose distribution resulted in an increase in the mean heart dose. The HBE plan significantly reduced the higher dose delivered to the heart, to a level similar to that achieved with the IMRT plan. Furthermore, it did not increase the volume affected by the low-to-moderate dose and the mean dose; this represents a significant advantage of the HBE plan.

RT may also result in pulmonary toxicity, ranging from acute radiation pneumonitis to chronic pulmonary fibrosis. The overall incidence rate of clinical radiation pneumonitis is 14%; a meta-analysis of breast cancer patients treated with 3D-CRT reported that 4% of patients developed high-grade radiation pneumonitis [29]. Numerous studies confirmed that the V20 of the ipsilateral lung is the most important parameter when considering pulmonary toxicity [30–33]. Lind *et al*. reported an increased incidence of radiation pneumonitis in patients with V20 > 20% compared with patients with V20 ≤ 20% (28.4% vs. 12.5%, respectively) [33]. The incidence of radiation pneumonitis was also significantly proportional to the mean ipsilateral lung dose [30, 33]. In this study, the dosimetric parameters mentioned above in the IMRT plan were similar to the upper dosimetric parameter limits of the previous studies. Furthermore, a distinct feature of the IMRT plan was its low dose distribution; in particular, the lung volume receiving 5–10 Gy was more than double that observed with the other plans. However, the effect of the low dose should not be overlooked. Pulmonary perfusion is diminished when more than 5–10 Gy is applied [34]. Wang *et al*. reported that V5 should be the cutoff point for predicting radiation pneumonitis. The incidence of radiation pneumonitis was only 3% in patients with V5 ≤ 42%, but, it was 38% in patients with V5 >42% [35]. The total lung volume receiving 10–20 Gy also correlated with a decrease in the carbon monoxide diffusing capacity and a decrease in pulmonary perfusion for 3 years in patients with breast cancer who were treated with radiotherapy [36]. Although the V20 of the ipsilateral lung satisfied the dose constraints of breast radiotherapy, changes in radiological findings and quality-of-life scores have been reported with V13 [37]. Elderly patients and those with underlying pulmonary disease, a history of lung resection, or a history of smoking are particularly vulnerable to radiation-related pulmonary toxicities [11, 13, 15, 38]. The incidence of grade 3 or higher radiation pneumonitis increased from 3% in lung cancer patients without interstitial lung disease to 26% in lung cancer patients with pre-existing interstitial lung disease [13]. Another study reported that the incidence of fatal radiation pneumonitis was 8.3% in patients with pre-existing interstitial lung disease compared with 0.35% in patients without interstitial lung disease (odds ratio = 22.6; *p* < 0.001) [15]. More rigorous dose limits for the lungs are therefore required to avoid severe pulmonary toxicity in vulnerable patients. The increased low dose distribution in the IMRT plan can cause pulmonary toxicity in these patients. In this respect, the 3D-CRT and HBE plans showed more desirable lung dose distributions compared with the IMRT plan.

Several other treatment techniques have been developed to reduce exposure of heart or lung during radiotherapy including prone positioning [39, 40], deep inspiration breath hold [41, 42], and proton beam therapy [43, 44]. If the deep inspiration breath hold is used concurrently with the HBE technique in this study, the shielded area of the heart block could be reduced and compromised dose coverage of the breast target volume could be also decreased.

In terms of target coverage, the best PTV coverage was seen with the IMRT plan in this study. Maximum OAR-sparing was achieved with the HBE plan, but this resulted in compromised PTV coverage. However, although the PTV coverage with the HBE plan did not reach the levels seen with the 3D-CRT or IMRT plans, the PTV V45, V47.5, and Dmean were 96.3%, 92.2%, and 50.6 Gy, respectively, which were within the respective acceptable ranges for whole breast radiotherapy. To evaluate the safety of a heart block, Raj *et al*. investigated the effect of complete heart block on local recurrence; the overall recurrence rate in patients who had undergone a complete heart block was similar to that of patients who had not undergone a complete heart block [45]. A higher incidence of local recurrence was only observed in patients in whom the heart block had occurred less than 2.5 cm away from the tumor bed (*p* = 0.05). Therefore, if tumor bed is not shielded by the heart block, the HBE plan represents a feasible alternative technique for patient at high risk for radiation-related toxicities.

In conclusion, this study showed that the HBE plan effectively reduced the amount of radiation exposure to the heart and ipsilateral lung. However, this is not a universal technique nor is it a complete substitute for the 3D-CRT or IMRT plans because heart block application leads to compromised PTV coverage. Significant critical organ-sparing takes priority over intact target coverage in some patients with severe underlying cardiac or pulmonary disease with a relatively low risk of local relapse. We suggest that the HBE technique should be considered at the time of treatment for select patients who may be vulnerable to radiation- related toxicities.
